# Is White Blood Cell Count Associated With Mortality in Peritoneal Dialysis Patients?: A Retrospective Single-Center Analysis

**DOI:** 10.7759/cureus.19728

**Published:** 2021-11-18

**Authors:** Koray Uludag, Tamer Arikan

**Affiliations:** 1 Department of Internal Medicine, Nephrology Division, Health Sciences University, Kayseri Medical Faculty, Kayseri City Hospital, Kayseri, TUR

**Keywords:** survival model, prognosis, peritoneal dialysis, mortality, white blood cell

## Abstract

Objective

White blood cell (WBC) count was used as a predictor in researches since it is a prognostic indicator and a substantial predictor of the development of cardiovascular disease (CVD). There have been very few reports looking at the association between WBC count and overall mortality in peritoneal dialysis (PD) patients. We intended to explore if the baseline total leukocyte count is linked to all-cause mortality, considering the association for linearity in PD patients.

Material and methods

The study comprised 204 incident PD patients who began treatment at the Nephrology Department of Health Sciences University, Kayseri Medical Faculty, Kayseri City Hospital between January 2009 and December 2017. The research period ended in January 2018. The link between baseline WBC count and all-cause mortality was studied using Cox proportional hazards models.

Results

The average age of the patients was 46.75 (8.49) years, and 48.5% were male. Diabetes and hypertension were prevalent in 59.8% and 76% of the population, respectively. The average WBC count was 9.37 (2.70) × 10^3^/µL. The mortality risk increased by 23% for every one-unit increase in the crude model. The hazard of death in the fully corrected model was 1.12 [95% confidence interval (CI): 1.02-1.23, p = 0.015]. In the models with WBC count stratified by tertiles, the mortality hazard of patients in tertile 2 was 2.38 (95% CI: 1.24-4.58, p = 0.009) and of patients in tertile 3 in the fully adjusted model was 2.64 (95% CI: 1.30-5.33, p = 0.007), compared with patients in tertile 1.

Conclusion

The initial WBC count may have a long-term impact on patient survival. Individuals with higher basal values or even an elevation in follow-up should therefore be strictly controlled, and all preventative measures should be made to lower the risk level.

## Introduction

Although there is an increase in the chance of survival in patients treated with renal replacement therapy (RRT) compared to the past, it is still not at the desired level. Therefore, much more predictive models need to be developed in this patient group to both prolong the lifespan and improve its quality [1,2]. Nearly half of the patients with end-stage renal disease (ESRD) die due to cardiovascular causes [3]. Although now well-known cardiac risk factors play a crucial role in this outcome, it is not wrong to think that other risks may contribute to this situation in patients with ESRD compared to the general population [4]. In this context, it is a known fact that malnutrition and inflammation are well-established prognostic factors in these patients [5,6]. As a result, it is of paramount importance to identify such risks early and prevent their progression, to achieve better clinical outcomes.

Complete blood count and white blood cell (WBC) count are thus inexpensive and widely used in clinics. WBC has been chosen as a variable in studies because it is a prognostic indicator and a significant determinant of cardiovascular disease (CVD) development [7,8]. Leukocytes release cytokines, which trigger inflammation and also increase the leukocyte count. Inflammation is the cause of many poor prognoses, including the development of heart failure [9,10]. In addition to the fact that the WBC count was defined as a determinant of adverse events in studies conducted in the general population [11,12], this property of WBC or its subtypes was also found in special groups such as patients receiving dialysis treatment or patients with predialysis stages [13,14].

Very few studies investigated the relationship between the WBC count and the mortality risk in peritoneal dialysis (PD) patients. In this context, in research conducted by Johnson et al., a possible nonlinear relationship between the WBC count and cardiovascular death was found, especially with the highest quartile of WBC count in 323 incident PD patients [15]. The fact that hemodialysis itself is an inflammatory trigger and the evidence supporting CRP decreases over time in PD patients suggests that further research is needed on whether WBC count in PD patients is independently associated with prognosis [16,17].

We aimed to investigate whether the basal total leukocyte count is associated with all-cause mortality in patients who receive PD therapy.

## Materials and methods

Setting and patients

This study was designed as a single-center retrospective observational cohort study. Consecutive patients who started PD in the nephrology department of Health Sciences University, Kayseri Medical Faculty, Kayseri City Hospital from January 2009 to December 2017 were included in the study. The follow-up time began on the date of entry into the cohort. Patients were observed until death, loss to follow-up, renal transplantation, transfer to another facility, or end of the study period of January 2018. The institutional review board approved this study with the exemption of the requirement for a written consent form. The exclusion criteria were (i) <18 years of age, (ii) a history of hemodialysis (HD) or kidney transplantation before PD (n = 19), (iii) active malignancy (n = 3), and (iv) dying within three months of the start of PD or failure to sustain PD for more than three months (n = 7). Ultimately, a total of 204 incident PD patients were included in the final analysis.

Variables

All-cause time-to-death was chosen as the primary outcome. Data from the hospital electronic records or patient files were used to gather information regarding mortality events, the date of the first dialysis treatment, demographic characteristics, body mass index (BMI), serum levels of hemoglobin, WBC count, ferritin, calcium, phosphorus, intact parathyroid hormone (PTH), total cholesterol, high-density lipoprotein (HDL), low-density lipoprotein (LDL), triglyceride, uric acid, C-reactive protein (CRP), albumin, sodium, and potassium. Baseline comorbidities considered were diabetes mellitus, hypertension, coronary artery disease, congestive heart failure, cerebrovascular disease, and peripheral vascular disease. All laboratory values were measured from fasting blood samples by automated and standardized methods. Blood samples were transported to the central laboratory center within 24 hours.

Cardiovascular disease was defined if the patients had a history of congestive heart failure, coronary, cerebrovascular, or peripheral vascular disease. We specified coronary artery disease when the patients had a history of angina, myocardial infarction, coronary angioplasty, or coronary artery bypass grafts. Cerebrovascular disease was defined as having experienced a transient ischemic attack, stroke, or carotid endarterectomy. Peripheral arterial disease was noted if a history of claudication, any peripheral revascularization procedure, or ischemic limb loss existed.

The modified peritoneal equilibration test was performed with 4.25% glucose dialysis. Furthermore, peritoneal transport characteristics were classified as high, average, and low based on the results of dialysate-to-plasma creatinine and glucose concentration ratios at four hours of dwell.

Residual renal function was measured one month after PD initiation using the average of the renal urea and creatinine clearances. Adequacy of dialysis was estimated by measuring total weekly Kt/V urea using standard methods. Peritoneal transport characteristics were determined using the equilibration ratios between dialysate and plasma creatinine.

Statistical analysis

Categorical variables were analyzed using the chi-square test and presented as frequencies and percentages. Continuous variables were evaluated using one-way ANOVA and the Kruskal-Wallis test. The results are presented as mean [standard deviation (SD)] for normally distributed variables and median (25th and 75th percentile) for variables with skewed distributions. WBC was divided into tertiles and analyzed as a categorical variable.

Cox proportional hazards models were used to determine the relationship of baseline WBC count with all-cause mortality and calculate hazard ratios and 95% confidence intervals. WBC levels were modeled as tertiles and continuous scale. Five models were considered: (i) unadjusted model (model 1), which included the main predictor; (ii) model 2, which included age and sex; (iii) model 3, which was adjusted for model 2 covariates + BMI, serum albumin, and hemoglobin concentration; (iv) model 4, which was adjusted for model 3 covariates + phosphorus, PTH, diabetes, hypertension, and cardiovascular disease; and (v) model 5, which was adjusted for model 4 covariates + glomerular filtration rate (GFR) and CRP level. The proportional hazards assumption was assessed using log-log plots and Schoenfeld residuals after model fitting. Data were analyzed using the R software version 4.0.3 for Windows. p-values less than 0.05 were considered significant.

## Results

A total of 204 patients met the inclusion criteria. The mean age was 46.75 (8.49) years, and 48.5% of the patients were male. The mean BMI was 25.49 (4.82), and the frequency of diabetes and hypertension was 59.8% and 76%, respectively. The prevalence of cardiovascular disease was 27.9%. The mean WBC levels were 9.37 (2.70) × 10^3^/µL. The median (p25, p75) follow-up time was 27.6 (13.2, 47.1) months, and 94 patients died during the study period. The demographic, clinical, and laboratory characteristics of the patients are shown in Table 1.

**Table 1:** Baseline characteristics of the PD cohort by white blood cell count tertiles. Data are presented as mean (SD), median [p25, p75], or n (%). p-values were estimated using chi-square or one-way ANOVA tests as appropriate. BMI, body mass index; PD, peritoneal dialysis; ACEI, angiotensin-converting enzyme inhibitor; ARB, angiotensin receptor blocker; eGFR, estimated glomerular filtration rate; PTH, intact parathyroid hormone; CRP, C-reactive protein; LDL, low-density lipoprotein; HDL high-density lipoprotein; CCB, calcium channel blocker

	Overall	Tertile 1	Tertile 2	Tertile 3	p
		(<8.2)	(8.2–10.5)	(>10.5)	
n	204	69	64	71	
Age, years	46.75 (8.49)	45.52 (8.87)	46.81 (7.33)	47.87 (9.02)	0.261
Male, n (%)	99 (48.5)	37 (53.6)	29 (45.3)	33 (46.5)	0.577
BMI, kg/m^2^	25.49 (4.82)	25.52 (5.04)	26.16 (4.63)	24.85 (4.76)	0.288
Diabetes mellitus, n (%)	122 (59.8)	33 (47.8)	35 (54.7)	54 (76.1)	0.002
Hypertension, n (%)	155 (76.0)	50 (72.5)	49 (76.6)	56 (78.9)	0.669
Cardiovascular disease, n (%)	57 (27.9)	16 (23.2)	18 (28.1)	23 (32.4)	0.478
Coronary artery disease, n (%)	23 (11.3)	5 (7.2)	8 (12.5)	10 (14.1)	
Congestive heart failure, n (%)	19 (9.3)	8 (11.6)	5 (7.8)	6 (8.5)	
Cerebrovascular disease, n (%)	9 (4.4)	1 (1.4)	4 (6.2)	4 (5.6)	
Peripheral vascular disease, n (%)	6 (2.9)	2 (2.9)	1 (1.6)	3 (4.2)	
Dyslipidemia, n (%)	104 (51.0)	37 (53.6)	32 (50.0)	35 (49.3)	0.862
Blood pressure > 140/90 mmHg, n (%)	33 (16.2)	11 (15.9)	8 (12.5)	14 (19.7)	0.523
Urine volume, L/day	596.00 [336.00, 906.00]	500.00 [281.00, 804.00]	617.50 [421.75, 958.00]	610.00 [353.50, 923.00]	0.164
PD ultrafiltration, L/day	914.50 [637.75, 1246.25]	1001.00 [609.00, 1272.00]	985.00 [694.50, 1293.50]	817.00 [648.00, 1066.50]	0.133
eGFR, mL/minute/1.73 m^2^	2.65 [0.88, 5.90]	3.90 [1.40, 7.00]	2.45 [0.80, 5.90]	1.70 [0.70, 4.85]	0.035
Total weekly Kt/V	1.90 [1.60, 2.40]	2.00 [1.70, 2.70]	1.95 [1.50, 2.50]	1.80 [1.55, 2.30]	0.233
Membrane transport type					
High, n (%)	25 (12.3)	12 (17.4)	5 (7.8)	8 (11.3)	0.231
Average, n (%)	163 (79.9)	51 (73.9)	52 (81.2)	60 (84.5)	0.279
Low, n (%)	37 (18.1)	13 (18.8)	8 (12.5)	16 (22.5)	0.314
ACEI/ARB use, n (%)	77 (37.7)	21 (30.4)	31 (48.4)	25 (35.2)	0.087
Diuretic use	36 (17.6)	12 (17.4)	7 (10.9)	17 (23.9)	0.141
CCB use, n (%)	89 (43.6)	34 (49.3)	28 (43.8)	27 (38.0)	0.406
Beta-blocker use, n (%)	43 (21.1)	18 (26.1)	12 (18.8)	13 (18.3)	0.455
Erythropoietin use, n (%)	84 (41.2)	23 (33.3)	32 (50.0)	29 (40.8)	0.149
Lipid-lowering drug, n (%)	56 (27.5)	15 (21.7)	24 (37.5)	17 (23.9)	0.090
Antiplatelet agent, n (%)	34 (16.7)	10 (14.5)	15 (23.4)	9 (12.7)	0.206
Phosphorus-lowering drug, n (%)	125 (61.3)	45 (65.2)	43 (67.2)	37 (52.1)	0.142
Vitamin D use, n (%)	73 (35.8)	24 (34.8)	24 (37.5)	25 (35.2)	0.941
White blood cell count, ×10^3^/µL	9.37 (2.70)	6.48 (1.39)	9.24 (0.67)	12.30 (1.41)	<0.001
Neutrophil count, ×10^3^/µL	6.53 (1.85)	5.48 (1.74)	6.29 (1.43)	7.76 (1.56)	<0.001
Lymphocyte count, ×10^3^/µL	2.17 (0.67)	1.77 (0.58)	2.13 (0.53)	2.59 (0.62)	<0.001
Monocyte count, ×10^3^/µL	0.70 (0.24)	0.54 (0.18)	0.69 (0.19)	0.86 (0.23)	<0.001
Hemoglobin, g/dL	11.28 (1.52)	11.71 (1.31)	11.25 (1.52)	10.89 (1.62)	0.006
Ferritin, µg/L	124.70 [83.22, 168.07]	111.70 [85.10, 161.30]	130.70 [76.55, 178.28]	133.40 [91.35, 184.00]	0.443
Serum creatinine, mg/dL	8.10 [5.70, 9.80]	8.40 [6.50, 10.70]	8.15 [5.53, 9.22]	7.30 [4.85, 9.30]	0.031
Serum sodium, mmol/L	138.92 (5.60)	138.60 (5.45)	139.44 (5.68)	138.75 (5.71)	0.658
Serum potassium, mmol/L	4.93 (1.79)	5.36 (1.80)	4.44 (1.81)	4.97 (1.67)	0.012
Calcium, mg/dL	9.11 (0.86)	9.22 (0.86)	9.10 (0.84)	9.01 (0.88)	0.353
Phosphorus, mg/dL	5.63 (1.07)	5.36 (0.98)	5.66 (1.15)	5.86 (1.02)	0.017
PTH, µg/L	171.25 [107.00, 264.17]	206.90 [115.20, 272.70]	174.00 [118.88, 260.50]	143.20 [83.70, 256.40]	0.194
Serum albumin, g/dL	3.51 (0.44)	3.67 (0.42)	3.51 (0.38)	3.36 (0.46)	<0.001
CRP, mg/L	5.30 [2.90, 9.07]	4.10 [1.80, 6.90]	4.70 [2.98, 7.20]	8.90 [4.40, 13.00]	<0.001
Uric acid, mg/dL	7.50 [6.38, 8.80]	7.30 [6.20, 8.50]	8.05 [6.90, 9.55]	7.50 [6.35, 8.50]	0.045
LDL, mg/dL	101.97 (19.44)	101.33 (19.66)	104.71 (18.23)	100.12 (20.26)	0.371
Triglyceride, mg/dL	183.75 [138.88, 247.15]	198.30 [149.30, 248.50]	181.70 [132.20, 256.95]	174.00 [137.45, 237.55]	0.591
HDL, mg/dL	45.64 (7.48)	46.11 (8.01)	45.89 (6.94)	44.96 (7.47)	0.628
Total cholesterol, mg/dL	188.65 (30.99)	189.14 (27.84)	191.00 (32.40)	186.04 (32.79)	0.643

When the relations with other variables were analyzed by dividing WBC into tertiles, some variables tended to increase or decrease with increasing WBC. WBC count was determined to be higher in patients with diabetes. In addition, WBC count increased significantly as serum phosphorus and CRP levels increased. Residual renal function, hemoglobin, serum creatinine, and albumin levels were found to decrease as the WBC count increased. It was evaluated that there might be a nonlinear relationship between serum potassium, uric acid levels, and WBC count.

When WBC count was modeled as a continuous variable and survival models were created, it was found that the mortality risk increased by 23% for every one-unit (10^3^/µL) increase in the crude model. In the models created by adding other confounding variables to the regression equation, it was determined that the statistical significance was maintained with the decrease in the effect level. Finally, the hazard of death in the fully corrected model was 1.12 (95% CI: 1.02-1.23, p = 0.015) (Table 2).

**Table 2:** Cox proportional hazard models for all-cause mortality according to WBC count as continuous scale and tertiles in peritoneal dialysis patients. The following adjustments were performed in regression models: model 1, crude model; model 2: model 1 + age and sex; model 3, model 2 + BMI, serum albumin, and hemoglobin; model 4, model 3 + phosphorus, PTH, diabetes, hypertension, and cardiovascular disease; model 5, model 4 + eGFR and CRP. WBC, white blood cell; Ref., reference group; HR, hazard ratio; CI, confidence interval

		Model 1			Model 2			Model 3			Model 4			Model 5	
	HR	95% CI	p	HR	95% CI	p	HR	95% CI	p	HR	95% CI	p	HR	95% CI	p
WBC, per 10^3^/µL increase	1.23	(1.14–1.33)	<0.001	1.22	(1.13–1.32)	<0.001	1.19	(1.10–1.29)	<0.001	1.14	(1.04–1.25)	0.004	1.12	(1.02–1.23)	0.015
WBC, categoric															
Tertile 1	Ref.			Ref.			Ref.			Ref.			Ref.		
Tertile 2	2.70	(1.45–5.04)	0.002	2.60	(1.39–4.86)	0.003	2.37	(1.25–4.47)	0.008	2.43	(1.27–4.67)	0.008	2.38	(1.24–4.58)	0.009
Tertile 3	4.49	(2.50–8.05)	<0.001	4.30	(2.39–7.74)	<0.001	3.48	(1.88–6.45)	<0.001	2.97	(1.50–5.87)	0.002	2.64	(1.30–5.33)	0.007

**Figure 1:**
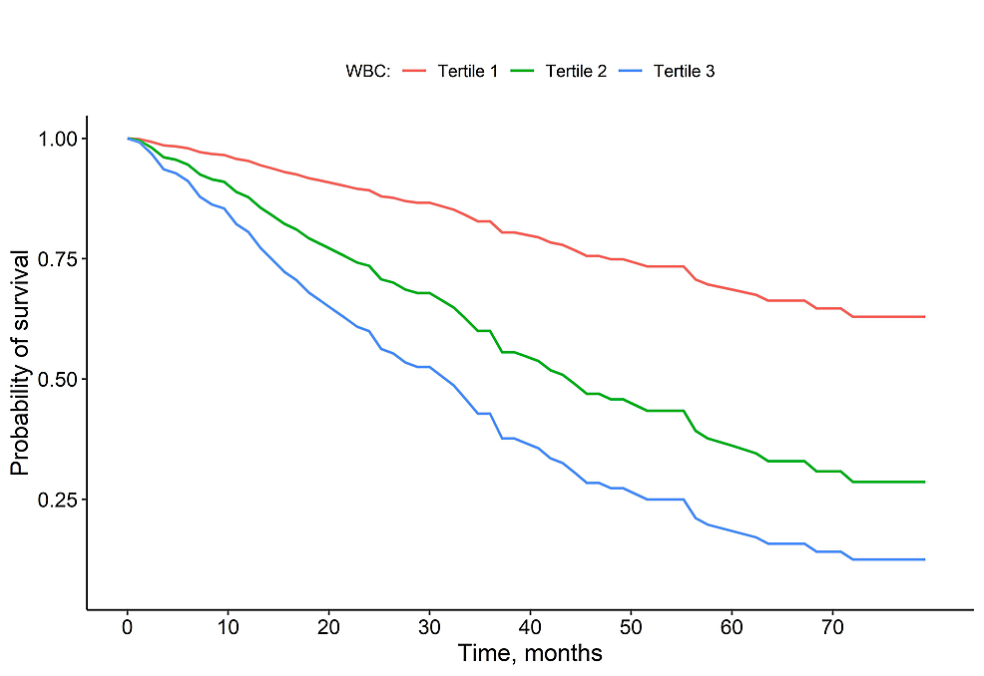
Kaplan–Meier curves for all-cause mortality based on the tertiles of the white blood cell count. WBC, white blood cell

**Figure 2:**
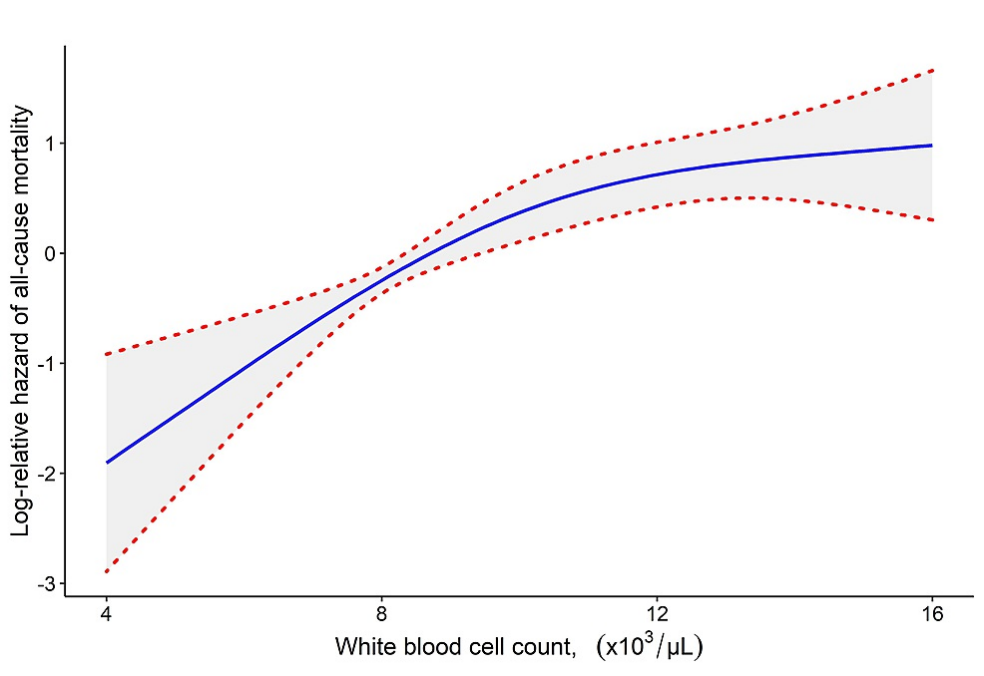
Unadjusted restricted cubic spline function with 3 degrees of freedom for the association between log-relative hazard of all-cause mortality and the white blood cell count (nonlinear p-value: 0.068).

When the models were repeated by categorizing the WBC count, the subjects in tertile 2 had 2.7 times higher risk of death compared with the patients in tertile 1. The patients in tertile 3 were 4.49 times more likely to die, according to the unadjusted model. Again, although there was a decrease in the level of effect when various adjustments were made, the mortality hazard of patients in tertile 2 was 2.38 (95% CI: 1.24-4.58, p = 0.009) and of patients in tertile 3 in the fully adjusted model was 2.64 (95% CI: 1.30-5.33, p = 0.007), compared with patients in tertile 1. Figure 1 shows the Kaplan-Meier plot showing the survival probabilities of WBC tertiles. Figure 2 shows the restricted cubic spline plot of the estimated relative mortality hazard obtained from the crude model according to the WBC count.

## Discussion

This analysis identified that the baseline leukocyte count predicts all-cause death in incident PD patients, regardless of potential confounding factors, suggesting that inflammatory episodes in PD patients have possible prognostic implications. Our results emphasize the need for screening WBC count and controlling the underlying reasons for WBC count increase in PD subjects. Also, we could conclude that our data would shed light on the pathophysiology of adverse events. Inflammation as a substantial independent risk factor for patient prognosis should be considered a target for intervention and prevention.

The association of WBC count with all-cause mortality was previously reported by Johnson et al. [15]. They found that patients in the top tertile had an increased risk of mortality, but not those in the lower tertiles, commenting that the relationship between WBC count and mortality may not be linear. We found that the rate of increase in the risk of death decreases after a certain point of WBC count in our study, but a statistically significant nonlinear relationship was not found between the two variables. Since our analysis was performed with only baseline values, it is necessary to take into account the confounding effect of intercurrent infections or a further increase in already existing inflammation intensity in this result. On the other hand, population-based studies reporting the prognostic importance of baseline WBC values will support our work. In the light of our findings, we could make the following interpretation that the risk of death increases linearly as the baseline WBC count increases.

Our data agree with earlier researches in the general and end-stage kidney failure populations, in which raised WBC count was established to be a robust and independent predictor of all-cause death or other prognostic outcomes. Reddan et al. determined that in a group of 44,114 hemodialysis patients, the neutrophil count was a significant independent risk factor for death on a survival model [18]. In a study of 959 maintenance HD subjects, researchers noted the WBC count strongly predicts one-year death from all causes, cardiovascular illness, and infection [19]. Kato et al. demonstrated that blood monocyte count was a determinant of cardiovascular and overall mortality in HD patients, while total WBC count did not vary substantially between dead and alive subjects [20]. In a multivariate-adjusted joint model, Agarwal et al. found that granulocyte and monocyte spikes were related to ESRD and mortality in 153 veterans without chronic kidney disease (CKD) and 267 with CKD [21]. A study looked at the prospective relationship between differential WBC count and incident heart failure events in 7195 healthy men and 8816 healthy women and found that inflammation, as defined by WBC count, was significantly linked with incident heart failure in healthy males, but not females [9]. Tsai et al. investigated the relationship between inflammation and renal prognosis in 3303 individuals with stage 3-5 CKD and revealed that WBC count was not associated with RRT or fast renal deterioration, contrary to our findings [22]. A recent study found that having a higher WBC count within the normal range was related to an increased risk of long-term death in 611 hemodialysis patients who began treatment at a tertiary hospital [23].

It is not fully understood why increased WBC leads to mortality. Although it has been suggested that smoking triggers the increase in WBC [24,25], it has been observed that the effect of WBC on mortality continues in statistical models where smoking and WBC are combined. On the other hand, when the models were adjusted with CRP or nutritional markers, which are indicators of systemic inflammation, the predictor effect of WBC continued in our study. However, we cannot definitively suggest a cause-effect relationship between increased WBC and mortality owing to the observational nature of the study. Some authors have reported that patients with CKD have macrophage and neutrophil infiltrates in the renal peritubular areas, and this has a negative correlation with GFR [26]. Perhaps the ongoing renal inflammation in PD patients worsens the prognosis by lowering residual renal function. Pfister et al. interpret that an independent relationship exists between the leukocyte count and the development of heart failure, apart from the general inflammatory response [9]. There is also evidence that granulocytes increase oxidative stress and enhance myocardial remodeling [27]. On the other hand, it has been reported that WBC has harmful effects by disrupting the antithrombotic and antiatherogenic properties of the endothelium [28]. Although such data were not available in the current study, such a process may engage in PD patients.

We should point out that there are some limitations to our study. First, the sample size was small, and the study was single-center and retrospective. Second, the measurement of variables was only at the baseline level. Third, there is the possibility that intervening episodes of infection or other unmeasured, residual confounding factors may have affected the study results. Fourth, generalizability cannot be performed because the study covers a specific geographic region. Finally, all-cause death was chosen as the endpoint due to the lack of cause-specific mortality data.

## Conclusions

In conclusion, the initial WBC count may have a long-term effect on patient survival, independent of other prognostic factors. Patients with a high baseline level or an increase in their follow-up should be followed more closely, and every measure should be taken to reduce the risk level.
